# Prevalence and correlates of sexually transmitted infections in pregnancy in HIV-infected and- uninfected women in Cape Town, South Africa

**DOI:** 10.1371/journal.pone.0218349

**Published:** 2019-07-01

**Authors:** Dvora L. Joseph Davey, Dorothy C. Nyemba, Yolanda Gomba, Linda-Gail Bekker, Sophia Taleghani, David J. DiTullio, David Shabsovich, Pamina M. Gorbach, Thomas J. Coates, Jeffrey D. Klausner, Landon Myer

**Affiliations:** 1 Department of Epidemiology, Fielding School of Public Health, University of California Los Angeles, Los Angeles, California, United States of America; 2 Division of Epidemiology and Biostatistics, School of Public Health and Family Medicine, University of Cape Town, Cape Town, South Africa; 3 Desmond Tutu HIV Foundation, Cape Town, South Africa; 4 David Geffen School of Medicine, UCLA, Los Angeles, California, United States of America; Centre Pasteur du Cameroun, CAMEROON

## Abstract

**Objectives:**

Sexually transmitted infections (STIs) are associated with adverse outcomes in pregnancy, including mother-to-child HIV transmission. Yet there are limited data on the prevalence and correlates of STI in pregnant women by HIV status in low- and middle-income countries, where syndromic STI management is routine.

**Methods:**

Between November 2017 and July 2018, we conducted a cross-sectional study of consecutive pregnant women making their first visit to a public sector antenatal clinic (ANC) in Cape Town. We interviewed women ≥18 years and tested them for *Chlamydia trachomatis* (CT), *Neisseria gonorrhoea* (NG) and *Trichomonas vaginalis* (TV) using Xpert assays (Cepheid, USA); results of syphilis serology came from routine testing records. We used multivariable logistic regression to identify correlates of STI in pregnancy.

**Results:**

In 242 women (median age 29 years [IQR = 24–34], median gestation 19 weeks [IQR = 14–24]) 44% were HIV-infected. Almost all reported vaginal sex during pregnancy (93%). Prevalence of any STI was 32%: 39% in HIV-infected women vs. 28% in HIV-uninfected women (p = 0.036). The most common infection was CT (20%) followed by TV (15%), then NG (5.8%). Of the 78 women diagnosed with a STI, 7 (9%) were identified and treated syndromically in ANC. Adjusting for age and gestational age, HIV-infection (aOR = 1.89; 95% CI = 1.02–3.67), being unmarried or not cohabiting with the fetus’ father (aOR = 2.19; 95% CI = 1.16–4.12), and having STI symptoms in the past three days (aOR = 6.60; 95% CI = 2.08–20.95) were associated with STI diagnosis.

**Conclusion:**

We found a high prevalence of treatable STIs in pregnancy among pregnant women, especially in HIV-infected women. Few women were identified and treated in pregnancy.

## Introduction

An estimated 357 million new cases of curable sexually transmitted infections (STIs) occur annually [1]. In low- and middle-income countries, the prevalence of curable STIs such as *Neisseria gonorrhoeae* (NG), *Chlamydia trachomatis* (CT), or *Trichomonas vaginalis* (TV) in pregnant women can be as high as 25% [1, 2]. Curable STIs are associated with increased adverse pregnancy outcomes such as miscarriage, stillbirth, and preterm birth [3–5]. Untreated STIs in pregnancy are associated with adverse outcomes in the neonate such as conjunctivitis, pneumonia, sepsis and infant death [3]. Studies have shown that risk of adverse events in pregnancy and neonates is increased when co-infections of two organisms are present [4].

In HIV-infected women, STIs increase the risk of mother-to-child transmission (MTCT) of HIV *in utero* and intrapartum [6]. In a recent study of HIV-infected pregnant women, other STIs nearly doubled the risk of having an HIV-infected infant [7]. Several mechanisms linking STIs with HIV transmission have been studied. Genital tract infections not only disrupt the protective mucosal barrier but increase the release of specific inflammatory cytokines linked to HIV acquisition [8, 9]. In HIV-uninfected women, STIs increase the risk of HIV acquisition [10, 11] and high viral load can significantly increase the risk of vertical HIV transmission [12].

Prior studies on the prevalence of curable STIs among pregnant women in Southern Africa report high prevalence of NG, CT, or TV, especially among women with HIV infection [8, 13, 14]. In one study among HIV-infected pregnant women in South Africa, almost half of women tested for CT, GC, or TV during their first ANC visit were positive for at least one organism and less than one-third reported symptoms [8]. Three studies reported higher prevalence of infection with CT only or CT as a co-infecting organism in HIV-infected pregnant women [8, 13, 14]. Another study among pregnant and postpartum women in South Africa who were HIV-infected or at high risk for HIV infection found that one-third tested positive for NG, CT, or TV at the first antenatal visit, half of whom reported symptoms [14].

A study of HIV-infected pregnant women in multiple sites including South Africa, Latin America, and the United States found that 20% of women were positive for CT and/or NG [4]. Higher rates of adverse infant outcomes (sepsis, pneumonia, conjunctivitis, death, low birth weight, or prematurity) were found among infants born to women with CT and NG co-infection (66%) versus with NG (57%), CT (39%) and no STI (37%) [4]. In most studies, including those previously reported from our group, a majority of pregnant women who tested positive for at least one STI were asymptomatic [8, 13, 14].

The combination of high STI prevalence, high proportion of asymptomatic infections, and adverse perinatal and neonatal outcomes, makes STI management a major public health concern [15]. Previous recommendations from the WHO of a syndromic approach to STI treatment during antenatal screening missed a large proportion of asymptomatic carriers. Without antenatal screening, the true burden of curable STIs in pregnancy remains uncertain [15]. In 2016, the WHO released its strategy report on ending STIs as a public health epidemic. The UN’s Global Strategy for Women’s, Children’s and Adolescents’ Health report states that 70% of countries should have STI surveillance systems in place by 2020, which requires a transition from syndromic management to etiologic surveillance, necessitating an increase in diagnostic capabilities [16].

Despite the strong evidence that STIs in pregnancy represent a major health challenge, few studies have assessed prevalence of curable STIs in HIV-infected and uninfected pregnant women receiving ANC including STI symptoms and syndromic management. Our study evaluates the prevalence of CT, NG, TV and syphilis in HIV-infected and uninfected pregnant women and identifies correlates of STI, including presenting symptoms, HIV infection status, age and partnership status.

## Methods

### Setting

Between November 2017 and July 2018, we conducted a cross-sectional study of pregnant women attending a public sector antenatal clinic (ANC) in Cape Town, South Africa. The clinic is a busy setting in a former township community outside of Cape Town. The population of 400,000 is predominantly of low socioeconomic status. The vast majority of the population uses local public-sector health services that are provided free of charge. In 2017, the HIV prevalence among women was 27%, similar to other clinics in and around Cape Town [17]. This site was selected because of our team’s ongoing research in maternal and child health in this setting.

### Eligibility and data collection

To be eligible for the study, women had to be ≥18 years and currently pregnant (<34-weeks) with the stated intention to reside in the community for the duration of the pregnancy. We hypothesized that STI prevalence would be higher in HIV-infected vs.–uninfected pregnant women. We enrolled 242 women based on sample size calculations of a cohort study that would detect a difference in prevalence of STIs between HIV-infected and uninfected women (estimated to be 18% in HIV-uninfected women vs. 33% in HIV-infected women or a risk ratio of 1.83) [2, 8]. After their routine first ANC visit, trained staff administered questionnaires investigating women’s socio-demographic background, sexual history during pregnancy, partner’s HIV status, prior STI treatment and recent STI symptoms (all self-reported). Data on HIV status was determined from maternal health records based on rapid HIV antibody testing administered at women’s first ANC visit as part of routine care. Interviews were conducted in a private room with a female interviewer who was trained to help decrease social desirability bias. Interviewers asked about intimate partner violence and if they reported it, participants were offered appropriate referrals. We evaluated prevalence of each STI by HIV infection status. We used multivariable logistic regression to identify factors associated with having a STI and then repeated the multivariable analysis controlling for *a priori* confounders.

### Specimen collection and testing

Women self-collected vulvovaginal swab specimens at first ANC, third trimester and post-partum visits, using Xpert CT/NG Vaginal/Endocervical Specimen Collection kits (Cepheid, Sunnyvale, CA), and trained study staff tested the swabs on site for CT, NG, and TV using the Xpert CT/NG assay and the Xpert TV assay (Cepheid). For this analysis, we report on the baseline visit (first ANC). Self-collected vaginal swabs have been shown to have a high sensitivity and specificity [18]. The Xpert CT/NG assay has a 99.5% sensitivity and 99.1% sensitivity for CT and 100% sensitivity and 99.9% specificity for NG. [19] The TV test has a sensitivity of 98.4% and 99.6% specificity in self-collected vaginal swabs.[19] The turnaround time for results was between 60–90 minutes. We reviewed the participant’s medical record to obtain results of syphilis testing using the national algorithm (rapid plasma reagent [RPR] followed by a Treponemal pallidum Haemagglutin Assay [TPHA] confirmatory test), which are part of standard antenatal care in South Africa [20]. The standard of care for STI screening and treatment is syndromic management, except for syphilis (20). Point of care (POC) testing for syphilis or other STIs is not yet available in the public sector in South Africa.

### Treatment

Women with a positive STI test result were treated in accordance with South Africa National guidelines [20]. During the study interview, women were asked if they had received treatment for their current symptoms. If they reported treatment in previous 2 weeks, we did not re-treat them. If they did not report treatment, women newly diagnosed were treated based on the National guidelines (89% received treatment same-day) (19). Women who were not treated on the same day as their diagnosis left the clinic instead of waiting the 60–90 minutes for the test results. Those women were treated within 1–2 days. According to the national guidelines, women who reported abnormal vaginal discharge/dysuria or vulva itching/burning received Ceftriaxone, IM, 250 mg as a single dose, and Azithromycin, oral, 1 gram, as a single dose and Metronidazole, oral, 2 grams as a single dose (20). Women who reported genital ulcers or sores with/without pain, or women diagnosed with syphilis were treated as part of the standard of care with intramuscular Benzathine penicillin given as 2.4 MU as a single dose (20); HIV-infected women with genital sores also received Aciclovir for genital sores/ulcers 400mg 8 hourly for 7 days. For their sex partner(s), women were counselled on the importance of partner testing and given a referral letter to attend the clinic for STI presumptive treatment. All women were provided condoms and counselling around condom use.

### Statistical analysis

We used univariate and bivariate analysis to describe participant characteristics. For categorical variables, frequencies and percentages are reported, and for continuous variables, medians and interquartile ranges (IQR) are presented. We used logistic regression models to evaluate the association between HIV status and STI in pregnancy. In addition, we evaluated secondary outcomes including socio-demographic and health factors associated with STI in pregnant women. We excluded syphilis in our analysis, as it was not part of our POC STI intervention, but was part of the standard of care. We adjusted for *a priori* variables in the models including mother’s age and gestational age, removing variables that were collinear in the final model (e.g. HIV status and couple’s HIV status). We calculated sensitivity and specificity of syndromic management of STIs using data on reported STI symptoms and treatment prior to study visit and during first ANC visit. Statistical analyses were performed using STATA version 14 software (StataCorp, 2015).

### Ethics

Ethical approval and oversight were provided by the Faculty of Health Sciences Human Research Ethics Committee at the University of Cape Town (#454/2017) and University of California Los Angeles (#19–000237). Written informed consent was obtained from all participants before enrolment.

## Results

We enrolled 242 pregnant women at first ANC visit. The median age of participants was 29 years (interquartile range [IQR] = 24–34 years). Half of mothers in the study reported being unmarried or not cohabiting with the father of their fetus, 47% reported being married or cohabiting with the father of their fetus, and 3% reported that they had no relationship with the father. Median gestational age was 19 weeks (IQR = 13–24). Overall, 44% of women enrolled were HIV-infected at first ANC (n = 107) and all but one reported being on ART after their first ANC visit, though 33% reported being newly diagnosed (n = 35). Approximately 3% of women reported intimate partner violence (IPV) in the past 12-months and 2% reported IPV during pregnancy. Approximately one-fifth (21%) of women reported ever having been treated for a STI in the past (Table 1).

**Table 1:** Sociodemographic, clinical and behavioral characteristics of pregnant women attending first antenatal clinic visit, Cape Town, November 2017-July 2018 (n = 242 women).

	n (%)
**Sociodemographic characteristics**	
Age (median, IQR)	29 (24–34)
Gestational age in weeks (median, IQR)	19 (14–24)
**Relationship with father of child**	
Married/Cohabiting	115 (47%)
Not married/ Non-cohabiting	120 (50%)
No relationship	7 (3%)
**Interpersonal violence (IPV)**	
Experienced IPV during past 12 months before pregnancy	8 (3%)
Experienced IPV during pregnancy	5 (2%)
**Clinical characteristics**	
Ever treated for STI	50 (21%)
Treated for STI in current pregnancy	78 (32%)
HIV infected	107 (44%)
HIV infected and on ART	106 (99%)
**Sexual behavior during pregnancy**	
Vaginal sex during pregnancy	225 (93%)
Fellatio sex during pregnancy	10 (4%)
Anal sex during pregnancy	7 (3%)
2+ sex partners in the past 3 months	3 (1%)
Suspected partner of having other sex partners	79 (33%)
**Partner’s serostatus**	
Concordant HIV negative	93 (38%)
Concordant HIV positive	34 (14%)
Discordant	26 (11%)
Don’t know partner’s status	89 (37%)

STI = sexually transmitted infection; IQR = interquartile range

### Sexual behaviors during pregnancy

Almost all women reported vaginal sex during pregnancy (93%). Of those women, 4% also reported fellatio and 3% reported having anal sex during pregnancy. No women reported forced sex in the past year before pregnancy or during pregnancy. One-third of women reported that they suspected their partner of having other sex partners and 1% reported ≥2 sex partners in the past 3-months.

### Partner’s HIV status

Over one-third of women did not know their partner’s HIV serostatus (37%) including 52 HIV-positive women (21%) and 38 HIV-negative women (16%). Thirty-eight percent reported being in a concordant HIV-uninfected relationship, 14% reported being in a concordant HIV-infected relationship and 11% reported that they were in serodiscordant relationships (4 women were HIV-negative with HIV-infected partners, while 27 were HIV-positive with HIV-uninfected partners). HIV-uninfected women were significantly more likely than HIV-infected women to report knowing their partners’ HIV status (72% vs. 52%; p = 0.002). Women who reported being in a concordant HIV-positive partnership had over twice the odds of having a STI in the unadjusted model (OR = 2.57; 95% CI = 1.14, 5.79; not included in multivariable model because of collinearity with participants’ HIV status).

### STI prevalence

**Fig 1:**
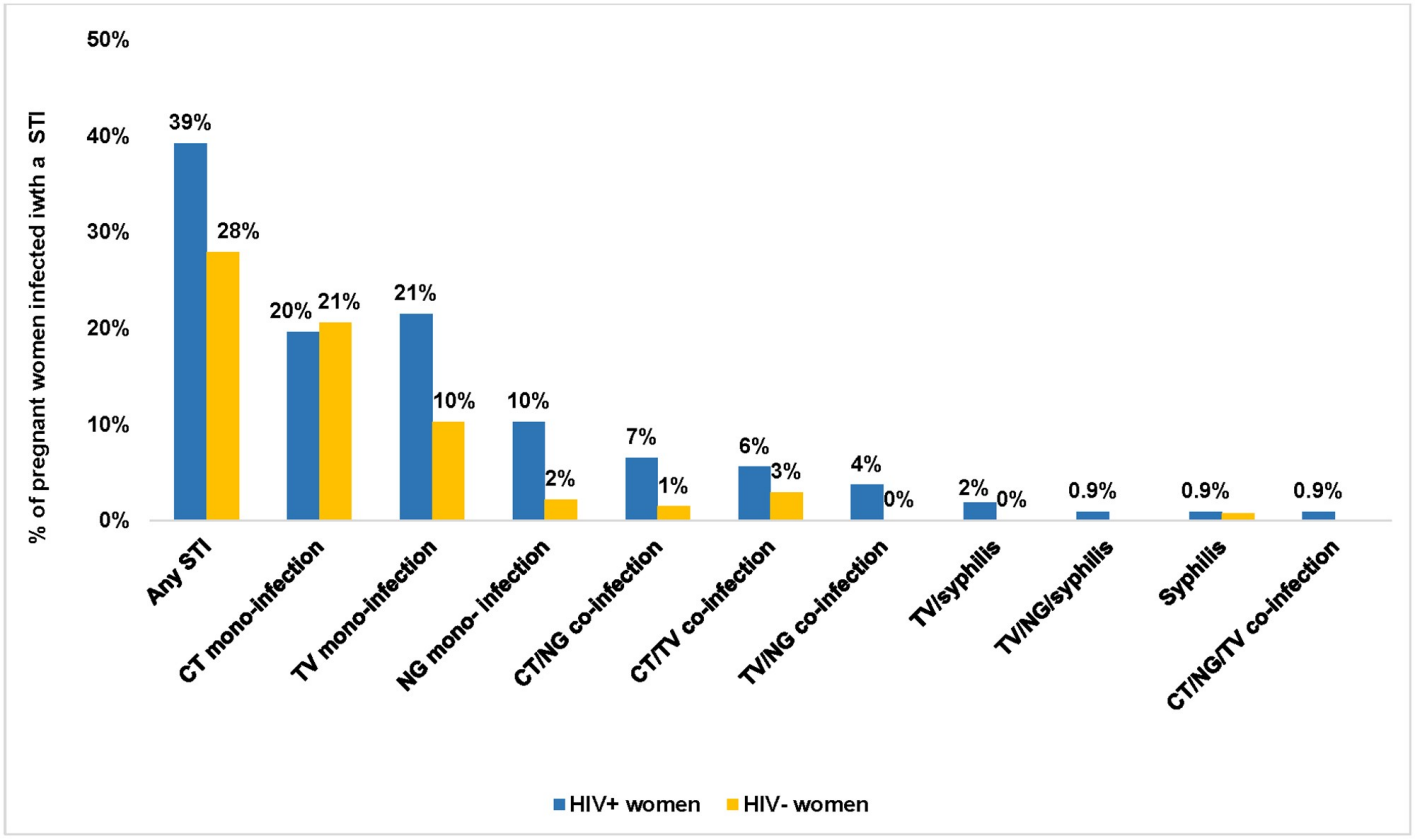
Prevalence of STI by type at first antenatal care visit in pregnant women in Cape Town, South Africa (n = 242). STIs include: *Chlamydia trachomatis* (CT), *Neisseria gonorrhoea* (NG) and *Trichomonas vaginalis* (TV).

Almost one-third of pregnant women were diagnosed with CT, NG and/or TV in our study (n = 78, 32%). In addition, five women had reactive RPR results (titer range of 1:4 to 1:256) for syphilis (of 232 women’s records = 2.2% prevalence). Three of the five women with syphilis had other STIs (2 women had TV and one woman had TV and NG), whereas two did not have another STI. The most common infection that we diagnosed was CT (20%) followed by TV (15%) then NG (5.8%). Overall 24 women had more than one infection: 3.7% had a CT/NG co-infection, 4.2% had a CT/TV co-infection, 1.7% had a TV/NG/syphilis co-infection, 2 women had a TV/syphilis infection (0.8%) and 1 woman had a CT/NG/TV co-infection (0.4%). Prevalence was higher in HIV-infected women for all STIs (p = 0.036) except for CT mono-infection (CT 21% in HIV-uninfected women vs. CT 20% in HIV-infected women) (Fig 1). Further, infection with more than one STI was more common in HIV-infected women. In HIV-infected pregnant women, 50% of those with a STI had more than one organism compared with 16% of HIV-uninfected women who were diagnosed with more than one organism (p<0.01).

### Sensitivity and specificity of symptoms in detecting STI

Of the 78 pregnant women who were diagnosed with one or more STIs at their first ANC visit (removing 2 women who were diagnosed with syphilis prior to study enrolment), 27 (35%) reported having had one or more STI symptoms during pregnancy, including abnormal discharge (n = 15), genital sores (n = 8), dyspareunia (n = 5), dysuria (n = 4), and vaginal bleeding (n = 3). Most reported having had a symptom more than a month ago (n = 11), or within the past three days (n = 11). Thirty-two women reported a symptom of a STI during pregnancy but did not test positive for CT, NG, TV or syphilis during this study (20%). Though 59 women reported symptoms of a STI only only 17 women (29%) reported being treated for a STI during this pregnancy (prior to study enrolment) of whom 7 were diagnosed with a STI (8.8% of 78 diagnosed). The positive predictive value of having any symptom and being diagnosed with a STI was 46% (95% CI = 34%, 58%) and negative predictive value was 72% (95% CI = 65%, 78%) (Table 2).

**Table 2:** Sensitivity, specificity, positive and negative predictive values with 95% confidence intervals of STI syndromic management and STI diagnosis, pregnant women, Cape Town, 2018.

		STI +	STI-	Total
Reported any STI symptom in pregnancy	**Yes**	27	32	**59**
	**No**	51	132	**183**
	**Total**	**78**	**164**	**242**
Sensitivity of syndromic STI management			35% (25%, 46%)	
Specificity of syndromic STI management			81% (74%, 86%)	
Positive predictive value			46% (34%, 58%)	
Negative predictive value			72% (65%, 78%)	

STIs include: *Chlamydia trachomatis* (CT), *Neisseria gonorrhoea* (NG) and *Trichomonas vaginalis* (TV); did not include prior syphilis diagnosis (n = 2 women)

### Factors associated with prevalent STI at first ANC

We present unadjusted estimates in Table 3. In an multivariable model adjusting for gestational age (adjusted odds ratio [aOR] = 1.03, 95% CI = 0.99, 1.08) and age (aOR = 0.95, 95% CI = 0.90, 1.00), being unmarried or not cohabiting with the father of the current pregnancy (aOR = 2.19, 95% CI = 1.16, 4.12), and being HIV-infected (aOR = 1.89, 95% CI = 1.02, 3.67), and reporting symptoms of a STI in the past three days (aOR = 6.60, 95% CI = 2.08, 20.95) were associated with being diagnosed with a STI (Table 3).

**Table 3:** Factors associated with sexually transmitted infection diagnosis (including participants diagnosed with syphilis) in pregnant women at first antenatal care visit (n = 242), Cape Town, 2018.

	Total, n (%)	Any STI, n (%)	No STI, n (%)	OR (95% CI)	Bivariate p-value	Adjusted OR (95% CI)*, **
Total	242	80 (33)^	162 (67)			
**Sociodemographic characteristics**						
Age (median, IQR)	29 (24–34)	28 (24–33)	30 (25–35)	**0.95 (0.91–1.00)**	**0.05**	**0.95 (0.90–1.00)**
Gestational age in weeks (median, IQR)	19 (13–24)	20 (14–24)	18 (13–23)	1.03 (0.98–1.07)	0.16	1.03 (0.99–1.08)
Relationship with father of child						
Married/Cohabiting	115 (47)	27 (35)	88 (53)	Reference		
Not married/ Non-cohabiting	120 (50)	47 (60)	73 (45)	**2.09 (1.19–3.69)**	**0.02**	**2.19 (1.16–4.12)**
No relationship	7 (3)	4 (5)	3 (2)	**4.34 (0.91–20.63)**	**0.06**	3.20 (0.63–16.09)
Ever experienced intimate partner violence	8 (3)	4 (5)	4 (2)	2.16 (0.52–8.88)	0.28	
**Clinical characteristics**						
Ever treated for STI	50 (21%)	13 (17)	37 (23)	0.65 (0.32–1.29)	0.21	
HIV Status						
HIV Negative	135 (56)	38 (28)	97 (72)	Reference		
HIV Positive	107 (44)	42 (39)	65 (61)	**1.65 (0.96–2.83)**	**0.07**	**1.89 (1.02–3.67)**
Any STI symptoms						
Yes	59 (24)	27 (35)	32 (20)	**2.18 (1.19–4.00)**	**0.01**	***
No	183 (76)	51 (65)	132 (80)	Reference		
Any STI symptoms (time period)						
No symptoms at all	183 (75)	51 (66)	132 (80)	Reference		
Symptoms in the past 3 days	16 (7)	11 (14)	5 (3)	**5.50 (1.82–16.61)**	**0.002**	**6.60 (2.08–20.95)**
Symptoms < 1 week ago	6 (3)	3 (4)	3 (2)	2.58 (0.50–13.24)	0.25	
Symptoms 1–2 weeks ago	4 (2)	1 (1)	3 (2)	0.86 (0.07–8.48)	0.89	
Symptoms 3–4 weeks ago	3 (1)	1 (1)	2 (1)	1.29 (0.11–14.58)	0.84	
More than a month ago	30 (12)	11 (14)	19 (12)	1.49 (0.66–3.36)	0.33	
Vaginal discharge						
Yes	35 (15)	15 (19)	20 (12)	1.71 (0.82–3.56)	0.15	
No	207 (85)	63 (81)	144 (87)	Reference		
Pain during intercourse						
Yes	13 (6)	5 (6)	8 (5)	1.33 (0.42–4.22)	0.63	
No	228 (94)	73 (94)	156 (95)	Reference		
Pain during urination						
Yes	15 (6)	4 (5)	11 (7)	0.75 (0.23–2.44)	0.63	
No	227 (94)	74 (95)	153 (93)	Reference		
Vaginal bleeding						
Yes	6 (2)	3 (4)	3 (2)	2.14 (0.42–10.88)	0.36	
No	236 (98)	75 (96)	161 (98)	Reference		
Genital sores						
Yes	8 (3)	5 (6)	3 (2)	**3.67 (0.85–15.79)**	**0.08**	
No	234 (97)	73 (94)	161 (98)	Reference		
Treated for STI symptoms this pregnancy						
Yes	17 (7)	5 (6)	12 (7)	0.86 (0.29–2.54)		
No	224 (93)	22 (28)	151 (93)			
**Sexual behavior during pregnancy**						
Vaginal sex during pregnancy	225 (93)	71 (90)	154 (94)	0.66 (0.24–1.80)	0.42	
Fellatio sex during pregnancy	10 (4)	3 (4)	7 (4)	0.89 (0.22–3.56)	0.87	
Anal sex during pregnancy	7 (3)	1 (1)	6 (4)	0.34 (0.04–2.89)	0.32	
2+ sex partners in the past 3 months	3 (1)	1 (1)	2 (1)	1.05 (0.09–11.77)	0.97	
Suspected partner of having other sex partners						
Yes	79 (33)	31 (40)	48 (29)	1.59 (0.89–2.80)	0.10	
No	162 (67)	47 (60)	116 (71)	Reference		
**Partner’s serostatus**						
Concordant HIV negative	93 (38)	26 (33)	67 (41)	Reference		
Concordant HIV positive	34 (14)	17 (22)	17 (10)	**2.57 (1.14–5.79)**	**0.02**	***
Discordant	26 (11)	6 (8)	20 (12)	0.77 (0.27–2.14)		
Don’t know partner’s status	89 (37)	29 (37)	60 (37)	-		

* bold is for p-value < 0.10

**Adjusted model included: mother’s age, gestational age, relationship status, HIV status, and symptoms of STI in past few days

*** Did not include in multivariable model because of collinearity with other variables

^ n = 80 participants diagnosed with *Chlamydia trachomatis*, *Neisseria gonorrhoea*, *Trichomonas vaginalis* and syphilis (n = 2 participants)

## Discussion

Our study identified high STI prevalence in both HIV-infected and -uninfected women attending their first antenatal visit at a community health center in Cape Town, similar to results found in previous studies of pregnant women in sub-Saharan Africa [2, 4, 8, 21]. Almost one-third of pregnant women tested positive for a common, treatable STI at their first visit. Of those who tested positive, 31% were co-infected with more than one STI. Odds of having a STI were higher in HIV-infected women, especially in partnerships in which the woman reported being in a concordant HIV-positive couple, consistent with research that suggests a complex relationship between HIV-infected women’s sexual behavior as well as their genital tract infections and increased risk of HIV acquisition [6–11]. Importantly, the vast majority of STIs diagnosed in participants were asymptomatic, and the sensitivity of syndromic management was only 35%. However, having a recent symptom in the past three days was associated with being diagnosed with a STI in the multivariable model after adjusting for age, gestational age and HIV infection status. This finding strongly suggests that though having a recent symptom was associated with STI diagnosis, syndromic management only identified one third of women with a STI, and left 52 pregnant women (65% of those diagnosed) without a proper diagnosis or treatment. This highlights the need to identify an appropriate screening tool to identify and treat STIs within this population of pregnant women. Without proper treatment of curable STIs, the risks of adverse events in pregnancy and in birth are increased (4–7). Furthermore, given over 90% of pregnant women remained sexually active during their pregnancy, partner notification and partner treatment are critical components of complete STI management to break the cycle of transmission.

Our findings re-emphasize that pregnant women in our study were high-risk for multiple STIs including HIV and syphilis, particularly young women. This could be due to HIV-infected women being at higher risk of STI acquisition; alternately, some women may have acquired STIs first, then HIV given the increased risk of HIV infection [22, 23]. Predictors of STIs such as younger maternal age have been demonstrated before [7]. Other predictors of STI diagnosis at first antenatal visit include being unmarried or in a non-cohabiting relationship, consistent with our previous work describing factors associated with high-risk behaviors during pregnancy [24].

Several prior studies have demonstrated a link between specific STIs with vertical transmission of HIV infection, highlighting the importance of STI screening in HIV-infected pregnant women who may not be on effective antiretroviral therapy or virally suppressed by the time they deliver [22, 23, 25–27]. In a recent evaluation by Adachi et al, women with STIs at birth had twice the risk of having an HIV-infected infant and risk was highest in women with two STIs. Similar to prior studies in pregnant women [4, 8, 25], we found that co-infection with more than one STI was common. In HIV-infected pregnant women, 50% of those with a STI had more than one organism, compared with 16% of HIV-uninfected women. The biological plausibility for STIs increasing the risk of HIV vertical transmission has been found in studies of non-pregnant women that demonstrated the association between STI and increased HIV genital shedding [28, 29].

HIV-uninfected women who have a STI are also at increased risk of HIV acquisition [10, 11]. Because the mechanisms of STI and HIV acquisition are so tightly linked, interventions developed to address primary HIV prevention in pregnancy should also address screening and treatment of STIs in pregnancy. In our study, up to one-third of women suspected their partner of having multiple partners, so a one-time POC test may be insufficient to protect women and their pregnancy. Rather, a combination of approaches that includes patient education, rapid and reliable point-of-care testing integrated into antenatal visits; and presumptive partner treatment with expedited partner therapy, should be developed to reduce the risk of HIV acquisition and vertical transmission in pregnancy. HIV-uninfected pregnant women who have STIs should receive information and access to pre-exposure prophylaxis (PrEP) to prevent HIV acquisition, which has been approved by the WHO for use in high HIV incidence communities [30–32]. However in South Africa, despite WHO guidelines, PrEP is contraindicated in pregnancy at the time of writing [32].

Given the high prevalence of asymptomatic STIs identified in both HIV-infected and HIV-uninfected pregnant women, numerous studies have commented on the discord between syndromic screening and point-of-care (POC) diagnostic testing. Previous studies in similar settings have found prevalence of STIs ranging from 21% to 54%, but only 14% to 28% of those who tested positive for an STI reported experiencing symptoms [33–36]. Furthermore, among studies in which it was reported, the ranges for additional diagnosis-related metrics for syndromic diagnosis of STIs were similar to that reported in our study, and they varied based on the STI and the symptom: sensitivity (9–66%), specificity (65–89%), positive predictive value (7–42%), and negative predictive value (55–85%) [8, 34, 37, 38]. While syndromic management may be more effective in other populations–pregnancy may increase the reporting of similar symptoms as an STI (vaginal discharge, pain during sex and urination [39]) which may mean that clinicians fail to screen effectively for these symptoms or women don’t report them because they are difficult to identify as unusual. The low sensitivity and low positive predictive value found in both our study and previous ones highlights the inadequacy of syndromic diagnosis of STIs in this setting and the need for more widespread use of more precise diagnostic methods. POC diagnostic methods for pregnant women has been shown to be cost-effective in high prevalence settings, such as South African clinics.

Our study provides an important foundation of evidence for increasing access to STI testing and treatment in this vulnerable population. However, the study does have some limitations. First, our study relies on self-reporting of study participants to collect sexual behavior data, which may be biased by recall or respondent bias. As a result, risky sex behaviors such as multiple partners may be under-reported. On the other hand, comparing self-reported data of STI symptoms to quantitative diagnostic methods provides important insight into how syndromic treatment is a poor predictor of STI diagnosis. Thus, continuing to collect self-reported data provides important insight into factors that must be understood to provide effective treatment interventions. Additionally, we only collected data from one facility which may limit generalizability to other populations; however, we selected a clinic with good representation of other clinics in the region.

Finally, our model identified several factors associated with increased STI prevalence, including younger age, being HIV-infected, as well as unmarried or in a non-cohabiting relationship. Pregnant women living with HIV infection are at risk of vertical and sexual HIV transmission and failure to diagnose and treat them for STI may increase the risk of onward vertical and sexual HIV transmission [4, 7, 22]. Given the poor sensitivity of syndromic management of STIs in pregnancy in our study, etiologic STI diagnosis is essential for this population. Those risk factors have important implications for partner notification strategies, as well as counseling of patients themselves, many of whom continue to engage in high risk behaviors during pregnancy [40].

## Conclusion

We found a very high prevalence of treatable STIs in pregnancy in pregnant women at first ANC visit in South Africa, especially in HIV-infected pregnant women. While having recent STI symptoms was predictive of STI diagnosis, syndromic treatment only led to a small proportion of women being treated successfully in this setting. Novel approaches to improve STI diagnosis and management in pregnancy are urgently required.

## Supporting information

S1 CRF (PDF)

S1 Dataset (DTA)
