# Supplementary material for: Prevalence and correlates of sexually transmitted infections in pregnancy in HIV-infected and- uninfected women in Cape Town, South Africa
Source: PLoS One. 2019 Jul 1;14(7):e0218349. doi: 10.1371/journal.pone.0218349 (PMC6602171; doi:10.1371/journal.pone.0218349)
Supplement: S1 CRF — (PDF) [file pone.0218349.s001.pdf]

Evaluating STI prevalence and incidence among pregnant and postpartum women in Cape Town, South Africa (STIP)

STIP CRF (ENGLISH)

To be completed by **ALL** enrolled STIP participants

Patient ID ______________

Visit Code ______

Visit Date							
D	D	M	M	Y	Y	Y	Y

We are now going to ask you some questions about your sex life. There are no right or wrong answers, please tell us about your personal experience.

A: Recent Sexual History	
1. How many sex partners have you had within the last three months?	__________________________
2. How many sex partners do you have now?	_________________________<br>***If 0 partners* -> SKIP to Q4**
3. What is your relationship status with the father of your child?	1. Married, living together<br>2. Married, not living together<br>3. Not married, living together<br>4. Not married, not living together
4. Is your most recent sex partner the father of your child?	1. Yes<br>2. No<br>3. Don't Know/Unsure
5. Have you had vaginal sex during pregnancy?	1. Yes<br>2. No
6. Have you had anal sex (when a man puts his penis in your anus) during pregnancy?	1. Yes<br>2. No
7. Have you had oral sex during pregnancy? Where you put your mouth on his penis?	1. Yes<br>2. No
8. Do you suspect your partner has other sex partners?	1. Yes<br>2. No<br>3. Don't know/Unsure
9. Is/was your partner's HIV status different from yours?	1. Yes<br>2. No<br>3. Don't know/Unsure

B: Knowledge, Attitudes, and Preferences regarding STIs

***Interviewer read**: "I am now going to ask you some questions about sexually transmitted infections or STIs."*

**<u>During this pregnancy have you had:</u>**<br>10. Abnormal vaginal discharge (green, yellow, foul-smelling)	1. Yes<br>2. No<br>3. Don't know/unsure
11. Increased pain during intercourse	1. Yes<br>2. No<br>3. Don't know/unsure
12. Pain during urination	1. Yes<br>2. No<br>3. Don't know/unsure
13. Vaginal bleeding	1. Yes<br>2. No<br>3. Don't know/unsure
14. Genital sores	1. Yes<br>2. No<br>3. Don't know/unsure
If no to questions 10-14, skip to Q21	
15. How many days or weeks ago did the symptoms start?	1. <1 week ago<br>2. 1-2 weeks ago<br>3. 3-4 weeks ago<br>4. More than a month ago<br>5. Don't know/unsure
16. Have you received treatment for the symptoms?	1. Yes<br>2. No ->**SKIP to Q18**
17. If yes, what kind?	☐ Bactrim<br>☐ Other: ________________
18. Did you notify your partner(s) of your symptoms?	1. Yes<br>2. No **-> SKIP to Q21**
19. What was your partner(s)' reaction when you notified them?	☐ 1. Willing to get tested or treated<br>☐ 2. Worried<br>☐ 3. Angry<br>☐ 4. Did not believe results<br>☐ 5. No reaction<br>☐ 6. Other ___________________
20. Did your partner(s) receive treatment?	1. Yes<br>2. No<br>3. Don't know/Unsure<br>(Probe if treatment is related to treatment received by participant)
21. <u>Before</u> this pregnancy, have you ever been told you have an STI by a healthcare professional?	1. Yes<br>2. No -> **SKIP to Q27**<br>3. Don't know/unsure -> **SKIP to Q26**
22. When were you told you had an STI?	1. Less than 12 months ago

	2. More than 12 months ago<br>3. Don't know/unsure
23. Did you receive treatment?	1. Yes **-> SKIP to Q25**<br>2. No
24. If no, why not?	1. I didn't want to<br>2. I could not afford it<br>3. My healthcare provider didn't offer me treatment<br>4. I was scared to ask my healthcare provider about my symptoms<br>5. Other ____________________<br>➔ **SKIP to Q26**
25. What were you treated for (check all that apply)?	***(Prompt)***<br>1. □ Abnormal vaginal discharge (green, yellow, foul-smelling)<br>2. □ Increased pain during intercourse<br>3. □ Pain during urination<br>4. □ Vaginal bleeding<br>5. □ Genital sores<br>6. □ Syphilis<br>7. □ Other ________________________<br>8. □ Don't recall
26. If you were found to have an STI, what would be your MOST IMPORTANT concern (select one)?	**(Do NOT prompt)**<br>1. Relieved to get it treated<br>2. Worried about my partner's reaction<br>3. Concerned about my partner's fidelity<br>4. Guilty<br>5. Worried about my health<br>6. Worried about my baby's health
27. If you were told you have an STI, who would be the FIRST PERSON who you would tell (select one)?	**(Do NOT prompt)**<br>1. I would not tell anyone<br>2. Partner<br>3. Family member<br>4. Friend<br>5. Healthcare worker<br>6. Teacher<br>7. Religious leader<br>8. Traditional healer<br>9. Spiritual healer
28. If you were told you have an STI, would you believe the result if you didn't feel/see any signs and symptoms?	**(Probe, but do not prompt)**<br>1. Yes, I understand I could have an STI but not feel the symptoms

	2. No, I would not trust the result, I would not take the treatment<br>3. No, I would not trust the result, but I would still take the treatment
29. Would you notify your partner if you tested positive for an STI in this study?	1. Yes<br>2. No<br>3. Not sure yet
30. Do you think your partner would be willing to take medication if you tested positive for an STI in this study?	1. Yes **->SKIP to Q32**<br>2. No<br>3. Don't know/Unsure **->SKIP to Q32**
31. If no, why do you think your partner would not want to get treated for an STI?	**(Do NOT prompt)**<br>1. He/she doesn't have time<br>2. He/she doesn't live close by<br>3. He/she doesn't like taking medication<br>4. Other ___________________
32. How would you prefer a vaginal swab to be taken (select one response)?	1. I would prefer to do it myself<br>2. I would prefer a nurse to do it for me<br>3. Either myself or a nurse can do it<br>4. I don't want to use a swab at all
33. Will you be able to wait in the clinic today to hear your results? This will take 60 minutes.	1. Yes **->SKIP to NEXT SECTION**<br>2. No
34. If no, why not?	1. I don't want to stay at the clinic that long<br>2. I have things I need to do<br>3. Other _____________________
35. How would you prefer to receive your results?	1. In person<br>2. Phone call<br>3. SMS/WhatsApp<br>4. Email<br>5. Other _____________________
36. Would you only like to receive your results if the results mean you will need to receive medical treatment?	1. Yes<br>2. No<br>3. Not sure

We are now going to ask you some questions about your relationship with your partner. Your answers will remain confidential from your partner, but if you feel uncomfortable answering any of these questions notify the study nurse.

C: RELATIONSHIP ASSESSMENT SCALE

**On a scale from 1, which is low, to 5, that is high please answer the following questions:**	Low				High
37. How well does your partner meet your needs?	1	2	3	4	5
38. In general, how satisfied are you with your relationship?	1	2	3	4	5
39. How good is your relationship compared to most?	1	2	3	4	5
40. How often do you wish you hadn't gotten into this relationship?	1	2	3	4	5
41. To what extent has your relationship met your original expectations?	1	2	3	4	5
42. How much do you love your partner?	1	2	3	4	5
43. How many problems are there in your relationship?	1	2	3	4	5

Hendrick, S. S. (1988). A generic measure of relationship satisfaction. *Journal of Marriage and the Family, 50,* 93–98.

These next set of questions ask about cases of intimate partner violence. If you feel uncomfortable by any of these questions, please notify the study nurse.

D: Intimate Partner Violence Questionnaire

	Q1. During the past 12 months before you became pregnant	Q2. Since you confirmed that you were pregnant	Q3. After you informed your partner of your STI result during the current pregnancy	Q4. During the current pregnancy, would you say this has happened once, twice, three times or more?
*has your **current** husband/partner ever….*				
1. insulted you, humiliated you, or threatened to hurt you?	44.<br>1. Yes<br>2. No	45.<br>1. Yes<br>2. No	46.<br>1. Yes<br>2. No	47.<br>1. 1 time<br>2. 2 times<br>3. 3 times<br>4. more than 3 times

hurt you physically (i.e. pushing, shoving, hitting, kicking) or used a weapon against you?	48.<br>1. Yes<br>2. No	49.<br>1. Yes<br>2. No	50.<br>1. Yes<br>2. No	51.<br>1. 1 time<br>2. 2 times<br>3. 3 times<br>4. more than 3 times
physically forced you to have sexual intercourse when you did not want to?	52.<br>1. Yes<br>2. No	53.<br>1. Yes<br>2. No	54.<br>1. Yes<br>2. No	55.<br>1. 1 time<br>2. 2 times<br>3. 3 times<br>4. more than 3 times
prohibited you from working or took your earnings if you had any income?	56.<br>1. Yes<br>2. No	57.<br>1. Yes<br>2. No	58.<br>1. Yes<br>2. No	59.<br>1. 1 time<br>2. 2 times<br>3. 3 times<br>4. more than 3 times

Signed Interviewer completing CRF: ___________________ Date: _____/_____/_________
DD MM YYYY

Signed QC Officer: _________________________________ Date: _____/_____/_________
DD MM YYYY

Signed Study Coordinator: _______________________ Date: _____/_____/_________
DD MM YYYY

F.15 Partner Notification Assessment Questionnaire

(To be completed ONLY at visits B and P for STI-positive women)

Visit Code ______

Patient ID ______________

Date (DD/MM/YYYY) _____________

We are going to ask you some questions about the notification letter we gave you at the last visit. Your answers will remain confidential and we encourage you to answer all the questions, but if any question makes you uncomfortable please notify the study nurse.

1. How many sex partners did you report during the last STIP study visit?	1. One<br>2. More than one: *complete additional questionnaire for each partner*<br>3. None: *end interview*
2. Did you give your partner(s) the notification letter within 7 days of your clinic visit?	1. Yes → *skip to #4*<br>2. No
3. If no, why not?	1. Partner was not home<br>2. Partner does not live clo se by<br>3. Partner does not have time<br>4. I did not think it was important<br>5. I misplaced the paper<br>6. I was afraid he will blame me<br>7. I was afraid of anger or abuse<br>8. I do not have a relationship with him<br>9. Other _________________<br>→ *skip to #11*
4. What was your most important motivation for notifying your partner?	1. Health of the baby<br>2. Health of my partner<br>3. My own health<br>4. Because the clinic staff recommended it<br>5. Other _________________
5. What was your partner's reaction when you gave him the notification letter?	1. Willing to get tested or treated<br>2. Worried<br>3. Angry<br>4. Did not believe results<br>5. No reaction<br>6. Other _________________
6. How did your partner treat you after you notified him?	1. Accused me of promiscuity or infidelity<br>2. Argued or fought with me<br>3. Refusal of sexual intercourse<br>4. Violence<br>5. Other _________________
7. Did your partner come to the clinic for treatment to your knowledge?	1. Yes<br>2. No → *skip to #10*<br>3. Not sure
8. Did he take the medication to your knowledge?	1. Yes<br>2. No<br>3. Don't know/ unsure

9. What do you think was his primary motivation to coming to the clinic for treatment?	1. Health of the baby<br>2. His health<br>3. My own health<br>4. Our health<br>5. The clinic letter<br>6. Other ________________<br>7. Don't know/ unsure
10. What was the most important barrier to your partner's coming in for treatment?	1. Work<br>2. Transportation<br>3. Time<br>4. Embarrassment/shame<br>Hesitant to come to a maternal obstetric ward<br>5. Other __________________<br>6. . Don't know/ unsure
11. How would you have preferred to notify your partner if given different options?	***Do not prompt***<br>1. Phone call from a health care provider<br>2. Text message from the clinic<br>3. Email message from the clinic<br>4. Give me treatment to give my partner<br>5. Would not notify<br>6. Other __________________
12. Would you prefer the notification be anonymous?	1. I would prefer anonymous notification<br>2. I do not prefer anonymous notification<br>3. I do not have a preference
13. Who have you told about your STI result (check all that apply)	1. ☐ No one<br>2. ☐ Partner<br>3. ☐ Family member<br>4. ☐ Friend<br>5. ☐ Healthcare worker<br>6. ☐ Teacher<br>7. ☐ Religious leader<br>8. ☐ Traditional healer<br>9. ☐ Spiritual healer<br>10. ☐ Other _____________________

Signed Interviewer completing CRF: _________________ Date: _____/_____/_________
DD MM YYYY

Signed QC Officer: ______________________________ Date: _____/_____/_________
DD MM YYYY

Signed Study Coordinator: _____________________ Date: _____/_____/_________

Appendix I: Partner Notification Letter

Date (DD/MM/YYYY): Patient ID:

Dear _________________,

This is a letter to inform you that your partner has been treated for the following STI(s);

☐ Chlamydia

☐ Gonorrhea

☐ Trichomonas

These are treatable sexually transmitted infections. Often people with these infections feel well and have no symptoms.

It is of the upmost importance that you receive treatment. Therefore, please bring this letter to the Gugulethu Midwife Obstetric Unit and ask for the study nurse within 7 days.

If you have any questions please call the study nurse:

Sincerely,
